# Swept Along: Measuring Otoacoustic Emissions Using Continuously Varying Stimuli

**DOI:** 10.1007/s10162-024-00934-5

**Published:** 2024-02-26

**Authors:** Christopher A. Shera

**Affiliations:** 1 Caruso Department of Otolaryngology, University of Southern California, Los Angeles, CA 90033 USA; 2 Department of Physics & Astronomy, University of Southern California, Los Angeles, CA 90033 USA

**Keywords:** Otoacoustic emissions, Swept tones, Chirps

## Abstract

At the 2004 Midwinter Meeting of the Association for Research in Otolaryngology, Glenis Long and her colleagues introduced a method for measuring distortion-product otoacoustic emissions (DPOAEs) using primary-tone stimuli whose instantaneous frequencies vary continuously with time. In contrast to standard OAE measurement methods, in which emissions are measured in the sinusoidal steady state using discrete tones of well-defined frequency, the swept-tone method sweeps across frequency, often at rates exceeding 1 oct/s. The resulting response waveforms are then analyzed using an appropriate filter (e.g., by least-squares fitting). Although introduced as a convenient way of studying DPOAE fine structure by separating the total OAE into distortion and reflection components, the swept-tone method has since been extended to stimulus-frequency emissions and has proved an efficient and valuable tool for probing cochlear mechanics. One day—a long time coming—swept tones may even find their way into the audiology clinic.

**Supplementary Information:**

The online version contains supplementary material available at 10.1007/s10162-024-00934-5.

## Introduction

When analyzing and interpreting otoacoustic emissions (OAEs), one endeavors to infer the status of the inner ear from indirect measurements. The problem has been likened to that of deducing the type and arrangement of the furniture in a closed room using only the light that leaks out from beneath the door [1]. This, of course, is a very hard problem. So, in most clinics, and in many research labs, OAE measurements are used only to address a much simpler question: Is the light switch on or off? Is hearing more or less normal, or is it not? Is it thumbs-up on the outer hair cells? Or thumbs-down?

The implication that otoacoustic emissions may only be useful for inferring the position of the light switch (on or off)—or, on days when one is feeling especially ambitious, to learn something about the wattage of the bulb—reflects a decidedly pessimistic view of the information that otoacoustic emissions carry back to the ear canal. The properties of otoacoustic emissions depend on much more than the health of the outer hair cells.

Of course, otoacoustic emissions *do* depend on the health of the outer hair cells. But they must also depend on a whole slew of other “parameters,” including round-trip middle-ear pressure gain, the value of the endocochlear potential, the operating point of the hair bundles, the strength of efferent feedback, the tilting angle of the OHCs, the height of the cochlear duct, and the form of the tonotopic map, to name only a few. One might imagine these many parameters as controlled by knobs. Different ears have different settings of the knobs. In one individual, the right and left ears may have relatively similar knob settings. Different individuals may have somewhat larger differences between the knobs. Still larger and more systematic differences may correspond to different species, or to different pathologies. In the analogy of the light leaking beneath the door, sitting in the room and idly turning the knobs will rapidly redecorate, fabricating a room different from the one you are in now.

The task of otoacoustic analysis can be regarded as using OAE measurements to deduce as much as possible about the settings of the knobs. In the strictest sense, of course, this task is entirely hopeless. There are simply too many knobs—and too few measurements—to reconstruct the setting of each and every one. But although one cannot deduce the setting of every knob, one might hope to infer large-scale patterns, or clusters, of knob settings that distinguish different individuals, different species, different pathologies.

Although the task appears daunting, we do have a soupçon of leverage. Ironically, much of this leverage comes about because OAE measurements come with their own imposing panel of knobs and switches. Unlike the physiological knobs introduced above, these knobs control parameters of the measurement and analysis protocols (e.g., the stimulus frequencies and levels, to name only the most obvious). In principle, they can be strategically manipulated by the investigator to maximize the information obtained. In addition to the control panel at our disposal, we can leverage the fact that different OAEs are different and do not all derive from the same source. Otoacoustic emissions are generated by at least two fundamentally different mechanisms within the cochlea [2], and these mechanisms—linear reflection and nonlinear distortion—depend on different and complementary aspects of cochlear mechanics [3, 4]. The existence of multiple stimulus control knobs and OAE source types suggests that it may prove profitable to ask whether otoacoustic emissions can tell us anything clinically useful about the room, other than whether the light is on or off. Answering this question, and subsequently translating the results to the clinic, requires many things, not the least of which is an efficient way to acquire the necessary otoacoustic data. By tackling this problem via the use of continuously varying (swept-frequency) stimuli, Glenis Long and her colleagues have made seminal contributions to improving the efficiency of OAE measurements.^1^ This review provides a simple, heuristic introduction to swept-frequency measurements, including conceptual summaries of their principal control parameters and analysis methods. Readers eager for technical details should consult the long reference list.

## Swept-Frequency Stimuli

**Fig. 1:**
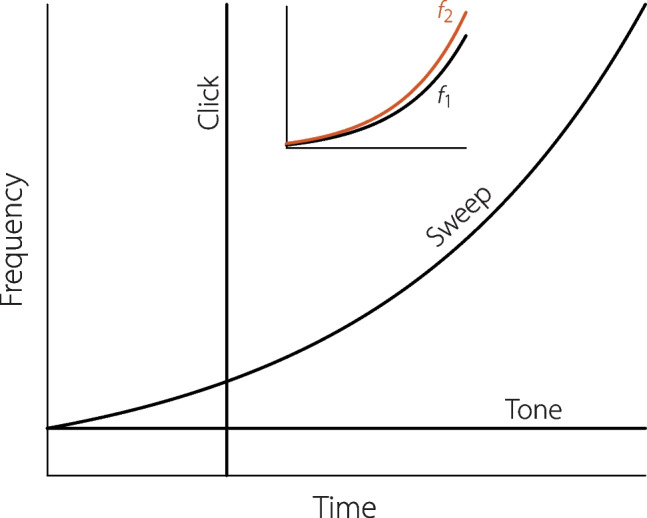
In the time-frequency plane, tones are represented by horizontal lines and clicks by vertical lines; sweeps appear as curves. The inset shows two exponential sweeps whose instantaneous frequencies, $f_1(t)$ and $f_2(t)$, remain in constant ratio

The acoustic stimuli used for auditory experiments, including the measurement of otoacoustic emissions, can be represented by curves that give the frequency (or frequencies) as a function of time (Fig. 1). For example, in 1978 David Kemp published the first report of otoacoustic emissions evoked by wideband clicks [9]. In the time-frequency plane, clicks appear as vertical lines, indicating that a broad range of sound frequencies is presented at a single moment of time. Stimulus-frequency OAEs, evoked by a pure tone, lie at the other extreme. In the time-frequency plane, pure tones appear as horizontal lines, indicating that a single sound frequency is presented over a broad range of times. The measurement of distortion-product OAEs, such as the cubic distortion product at frequency $2f_1-f_2$, usually involves the presentation of two or more pure tones, each of which would again be represented by a horizontal line.

In contrast to the rigid orthogonality of clicks and tones, swept-frequency measurements take advantage of the freedom one has to vary stimulus frequencies continuously over time. In the time-frequency plane, sweeps appear as sloping curves, and are thus in some sense intermediate between clicks and tones. The two sweeps shown in the inset of Fig. 1 might be used to evoke distortion-product OAEs; their instantaneous frequencies $f_1(t)$ and $f_2(t)$ start out low and increase exponentially over time while remaining in constant relative ratio.

**Fig. 2:**
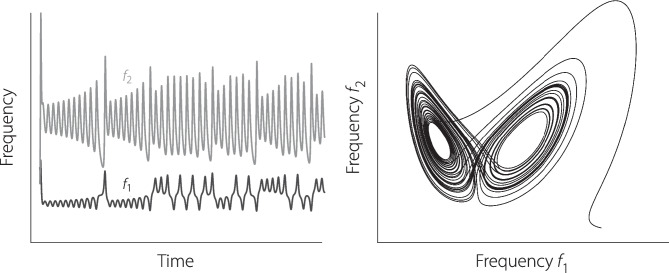
Left: Simple example of two nonmonotonic frequency sweeps, $f_1(t)$ and $f_2(t)$. Right: Deterministic nonperiodic trajectory that emerges when $f_2(t)$ and $f_1(t)$ are plotted against one another (see Online Resource 1)

Nothing requires that sweep trajectories be monotonic or traversed at a uniform rate [10]. For example, $f_1(t)$ and $f_2(t)$ can easily be varied in more complex and interesting ways, such as those shown in Fig. 2. (You can listen to this strangely attractive stimulus by consulting Online Resources 1 and 2.) To identify the sound, it may help to see the trajectory that emerges when $f_2(t)$ and $f_1(t)$ are plotted against one another (right-hand panel). In case you do not immediately recognize the resulting trajectory, it represents the solution to a simplified mathematical model of atmospheric convection devised by Edward Lorenz at MIT in the early 1960s [11].

**Fig. 3:**
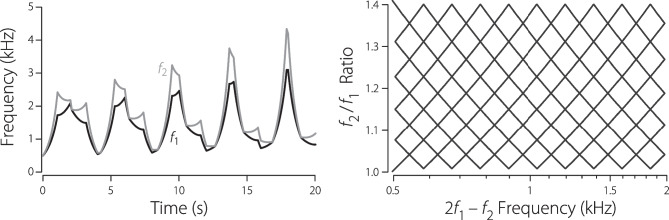
Left: Two nonmonotonic frequency sweeps, $f_1(t)$ and $f_2(t)$, with potential application to DPOAE measurements [15]. Right: Sawtooth “Lissajous” trajectory that emerges when the ratio $f_2(t)/f_1(t)$ and is plotted against the frequency of the cubic distortion product $f_\textrm{DP}(t)=2f_1(t)-f_2(t)$. The sweep stimulus begins in the lower left-hand corner (near $\{f_\textrm{DP},f_2/f_1\}=\{0.5,1\}$) and ends some 20 s later in the upper left (near $\{0.5,1.4\}$). For more details, consult Online Resources 1 and 3

Although the time-frequency trajectories shown in Fig. 2 help highlight the vast library of compelling swept-frequency waveforms, the otoacoustic utility of these particular stimuli remains unresolved. Figure 3 provides an example with clearer application to the measurement of DPOAEs. The left-hand panel shows the primary frequencies $f_1(t)$ and $f_2(t)$, each of which initially appears as a curious concatenation of up- and down-sweeps with perhaps some internal rhyme but little reason. The hidden structure appears when the frequency ratio $f_2(t)/f_1(t)$ is plotted vs the frequency of the cubic distortion product $f_\textrm{DP}(t)=2f_1(t)-f_2(t)$. This representation reveals that the stimulus provides a means of measuring high-resolution DPOAE $f_1,f_2$ “area maps” of the sort pioneered by Knight and Kemp [12, 13] for the study of “wave-” and “place-fixed” generation mechanisms. Conventionally explored by using a series of discrete tones at various $f_2/f_1$ ratios, the desired stimulus space can also be traversed, as demonstrated here, using an analogue of the “Lissajous” protocol [7] developed to efficiently span the $L_1 \times L_2$ level space using sweeps of continuously changing intensity. (You can use this stimulus to evoke DPOAEs from your own ears by listening to Online Resource 3.) Although considerably less baroque than this example, sweeps strategically constructed to vary the $f_2/f_1$ ratio optimally over time can help to maximize DPOAE levels across frequency [14].

### The Broad Sweep of History

**Fig. 4:**
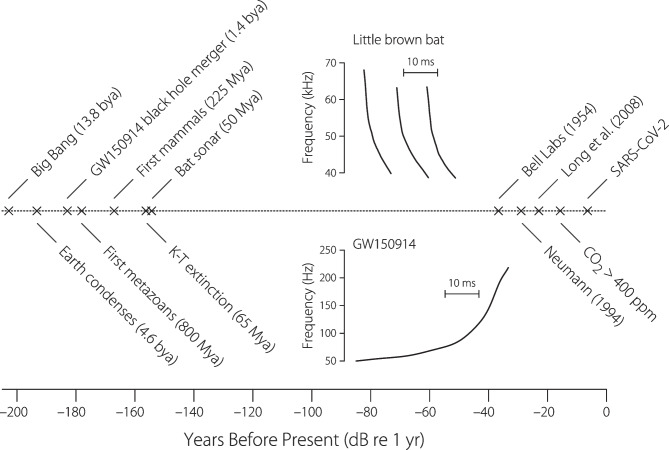
The long history of swept-frequency signal processing. Time before the present (here taken to be Monday, February 7th, 2022) appears along the abscissa in dB re 1 year [i.e., $20\log _{10}(\Delta t/[\textrm{yr}])$]. The upper inset shows an example spectrogram of the swept-frequency, echolocating call of the little brown bat. The lower inset shows the instantaneous frequency of the chirp-like gravitational wave launched by the inspiralling merger of two $\sim$30 solar-mass black holes during the final 40 ms before their coalescence more than a billion years ago [16, 17]. The outgoing wave of spacetime distortion from this collision swept past the Earth at approximately 5:51 am Eastern Standard Time on September 14, 2015. (The event is known as GW150914 for the date of it detection by the twin observatories of LIGO.) Note that the gravitational wave and the chiropteran chirplets share a similar time scale ($10\,$ ms horizontal scale bar)

Even if Glenis had never been involved, swept-frequency measurements would have a long history. Figure 4 shows a timeline in which years before the present are given in dB relative to 1 year. Thus, going right to left, each increment of 20 carries us another factor of 10 into the past.^2^ Exploiting chirps to make precision acoustic measurements was pioneered by bats, where it evolved some 30–50 million years ago.^3^ By evolutionary standards, this was not too long after the Cretaceous-Tertiary extinction swept away the dinosaurs and cleared the way for the rise of mammals, eventually including those of us who, like Glenis, share a special fondness for bats. After that, there is a sizable longueur on this logarithmic time scale during which little new happens, although the night skies presumably resounded with ultrasonic chirping, as shown in the upper inset. Finally, after another 25 million years, we humans became involved when on the heels of World War II radio engineers at Bell Labs began filing for patents. Neumann and colleagues [21], and later Keefe [22], published the first applications to the measurement of otoacoustic emissions. Long, Talmadge, and Lee published their first paper in 2008 [23], and many other groups have since been swept along.

### Sweep Rates Fast and Slow

Perhaps the most important control knob of any sweep stimulus is its rate, which determines how fast the frequency changes with time. The sweep rate is defined as the instantaneous slope of the frequency-vs-time curve. When expressed in Hz/s, the rate of an exponential sweep increases (or decreases) continuously; when expressed in octaves/s, the rate is constant.

How does one know where to set the knob? The sweep rate of a tone is zero, and the sweep rate of a click is effectively infinite. Clearly, there is no shortage of possible values in between. For guidance on setting the knob, it would help to know whether a given rate should be considered “fast” (i.e., more like a click) or “slow” (more like a tone). But how can one decide if any given sweep is fast or slow? Since nothing is great or little otherwise than by comparison [24], we need a meaningful reference standard, one with physical or biological significance.

One possibility for distinguishing fast from slow is to compare the sweep rate with the speed (or group velocity) of the traveling wave near its peak. This velocity can be expressed in appropriate units, such as octaves/s, using the tonotopic map. The idea is that if the sweep traverses the region of emission generation much more slowly than the traveling wave, then the cochlear response approximates the steady state, and the sweep can therefore be regarded as “slow.” At the other extreme, when the sweep moves across frequency much more rapidly than the traveling wave, then the cochlear response is transient in nature, and the sweep is therefore “fast.”

**Fig. 5:**
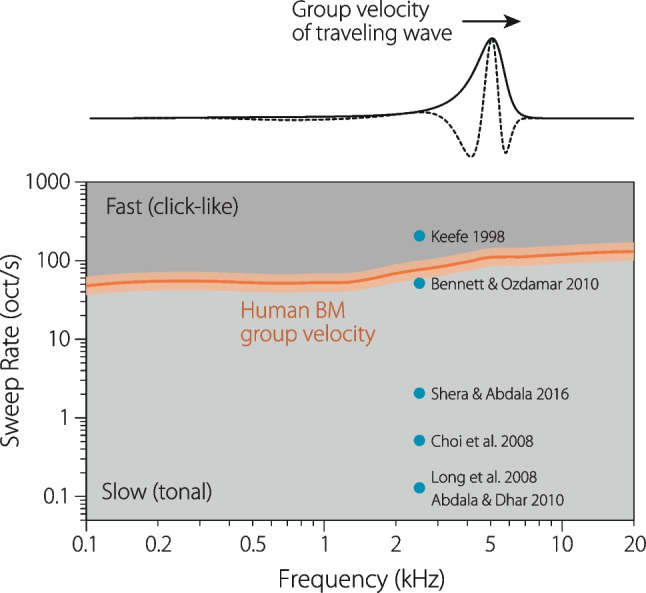
The group velocity of the basilar-membrane (BM) traveling wave near its peak divides the rate-vs-frequency plane into regions corresponding to fast (click-like) and slow (tonal) sweeps. The dark orange line (with flanking uncertainty bands spanning $\pm 30$%.) provides an estimate of human BM group velocity derived from OAE measurements (see note 4). The blue dots, arbitrarily placed at 2.5 kHz along the abscissa, show the sweep rates employed in representative OAE studies [22, 23, 25–28]

In Fig. 5, the dark orange line and its flanking uncertainty band provide an estimate of the traveling-wave group velocity for the human cochlea.^4^ The group velocity varies somewhat with frequency (and also with sound level) but is typically about 50–100 octaves/s. The line divides the plane into two regions. Sweep rates above the line evoke transient responses in the cochlea and can be considered fast (or click-like). We might call them “chirps” to distinguish them from swept tones. Sweep rates that fall below the line are slow (or tonal). Shown for comparison, the sweep rates adopted by a handful of representative studies run the gamut from slow to fast [22, 23, 25–28]. The figure also emphasizes the principal contribution made by Glenis and her colleagues, which was to pioneer the use of *slow* sweeps.

For simplicity, most OAE studies have employed constant sweep rates (whether in Hz/s or oct/s). But Fig. 5 suggests that employing faster rates at higher frequencies (i.e., using variable sweep rates that parallel the BM group velocity curve) might be more natural, perhaps helping to compensate for place-specific changes in cochlear mechanics or OAE generation, such as the well-known differences in scaling behavior between the apical and basal regions of the cochlea [31–35]. Reducing the sweep rate at low frequencies can also be a useful tool for countering the frequency dependence of contaminating noise sources. Since biological and environmental noise is generally more troublesome at lower frequencies, adjusting the sweep rate to increase the “dwell” or effective measurement time at these frequencies—or, more generally, as a function of the expected noise floor—can help to ensure a more uniform SNR across frequency [10].

Chirps and swept tones have different strengths and weaknesses for measuring OAEs, and they often require different analysis methods. But does the choice of sweep rate, fast or slow, affect the actual emission generated by the cochlea? Answering this question remains an active area of research, but for reflection-source emissions, the answer appears to be “not too much” [36, 37]. This is consistent with the observation that basilar-membrane frequency responses measured using clicks are remarkably similar to those obtained using tones of matched intensity [37, 38]. The effect of sweep rate on distortion-source OAEs has not been well studied. Part of the reason may be that it is simply harder to know how to make the comparison. (What, after all, is $2f_1-f_2$ for a click?) We will return to this issue when discussing the effect of sweep direction on DPOAEs. The remainder of this review will follow Glenis’ long trail and focus on slowly swept tones, using the measurement of DPOAEs as an example.

## Measuring Swept-Tone OAEs

**Fig. 6:**
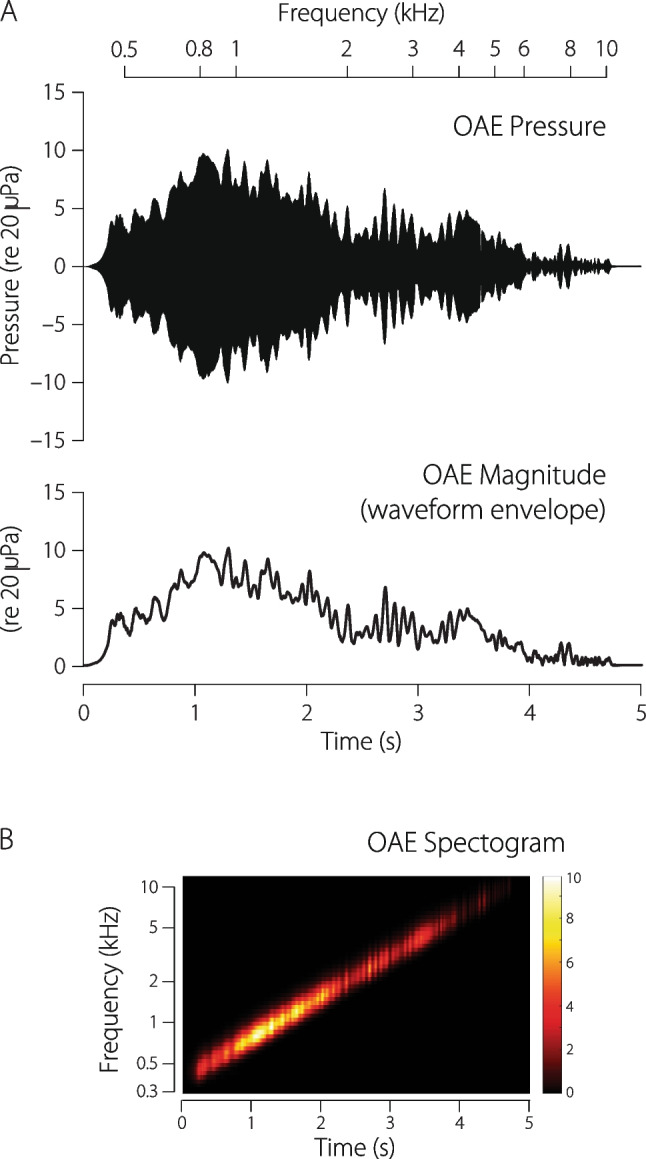
Example DPOAE waveform measured using swept-tone stimuli in a human ear. The top half of panel A shows the OAE pressure residual obtained when using phase-rotation averaging to cancel out the primary tones at frequencies $f_1$ and $f_2$. The waveform envelope appears immediately below. Panel B shows a time-frequency representation of the OAE pressure waveform. The color scale gives the OAE pressure in units of 20 $\mu$Pa. Sweep parameters: $\textrm{rate}=1\,$ oct/s, $f_2/f_1=1.22$; $\{L_2,L_1\}=\{55,61\}\,$ dB FPL

Figure 6A shows the time waveform of the distortion-product OAE evoked by an exponential sweep (top panel). The stimulus tones at $f_1$ and $f_2$ were swept upwards at 1 oct/s for about 5 s, and the primary tones were removed from the measured response using phase-rotation averaging [39]. The resulting residual is dominated by the cubic distortion component at frequency $2f_1-f_2$. Although it can be difficult to appreciate by simply looking at the waveform, the spectrogram in Fig. 6B makes it clear that the emission frequency is indeed changing over time. Converting the time axis to frequency using the sweep rate shows that the DPOAE frequency increases exponentially, covering almost 5 octaves, from about 400 Hz to around 10 kHz.

How does one estimate the OAE magnitude and phase from this waveform? One easy way to extract the OAE magnitude is to compute the waveform envelope (Fig. 6A, bottom panel). The ripples in the envelope reveal the presence of DPOAE fine structure, which results from interference between the distortion and reflection components [40–42]. Although estimating OAE magnitude by computing the waveform envelope works well in this example, the method is not especially robust. It yields reliable results only when measurement noise and other contaminants are small. The method also provides no estimate of the emission phase.

**Fig. 7:**
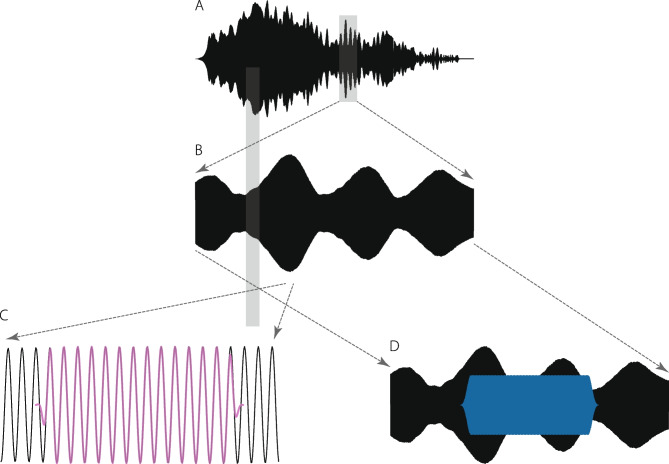
Estimating OAE magnitude and phase using least-squares fitting. The analysis begins by zooming in on a segment of the measured pressure waveform (panels A, B). The total DPOAE (magnitude and phase) can be obtained by fitting swept-frequency tone pips of matching sweep rate to short, windowed segments of the pressure waveform (panel C). By analyzing short segments, one captures rapid modulations in OAE amplitude (e.g., the fine-structure ripples). The magnitude and phase of the slowly varying distortion component can be obtained by fitting to longer windowed segments (panel D), which smooths by averaging over the spectral ripples (i.e., filters out the long-latency component, whose phase varies more rapidly)

### Least-Squares Fitting

**Fig. 8:**
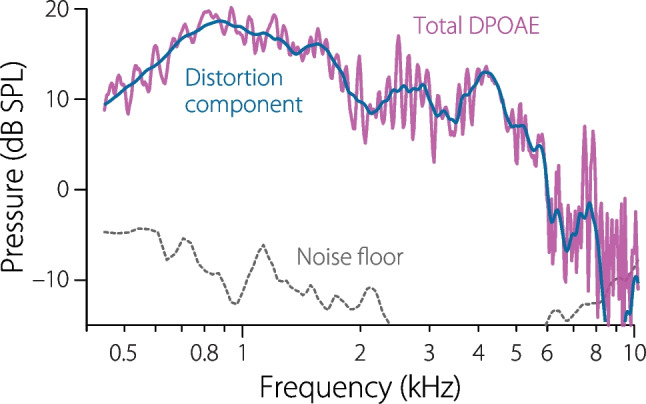
DPOAE magnitude spectra obtained by fitting a swept-frequency tone-pip to windowed segments of the measured pressure waveform (see Figs. 6 and 7) using the least-squares method. The total DPOAE (purple) was obtained using a short analysis window corresponding to a narrow frequency band (here, spanning $\sim$ $\frac{1}{4}$ of a ripple period); the distortion component (blue) was obtained using a longer window spanning a wider frequency band ($\sim$ $1\frac{1}{2}$ ripple periods). The dotted line shows the measurement noise floor. The least-squares method also provides estimates of OAE phase (not shown)

A better method would take advantage of the fact that we know the emission frequency (i.e., the value of $2f_1-f_2$) at each instant of time. With this in mind, Glenis and her colleagues applied a procedure based on fitting a mathematical model to segments of the measured waveform using least squares [23]. Figure 8 illustrates how it works. Zooming in on the shaded portion of the waveform in panel A reveals the rippled segment appearing in panel B. To extract the OAE at a particular frequency—say, at the local maximum in the fine structure (narrow shaded region)—one locates the corresponding time in the waveform when the frequency $2f_1-f_2$ passes through this frequency. Zooming in further around this point, we obtain the waveform snippet reproduced in panel C. The emission magnitude and phase at the center frequency of the window can then be estimated by using a least-squares procedure to fit a swept-frequency tone-pip of matching instantaneous frequency and rate to the waveform segment. The result is illustrated in purple. Repeating this fitting procedure at a large number of different times (or, equivalently, frequencies), yields an estimate of the entire DPOAE spectrum. The spectrum shown in Fig. 8 was computed at frequencies separated by one musical cent (1/100 of an octave). One can obtain an estimate of the effective noise floor of the measurement and analysis procedure (dotted line) either by analyzing the same waveform slightly “off-frequency” or by analyzing multiple sweeps “on-frequency” and computing the standard error of the mean.

### Unmixing the Components

To remove the fine structure, and thereby unmix the distortion and reflection components, one can perform the least-squares fitting using an analysis window that spans a longer segment of the time waveform and thus a wider band of frequencies (see Fig. 7D). The blue segment spans a frequency range of about one and a half fine-structure (ripple) periods. In this case, the fitting procedure yields an estimate of the DPOAE in which the rapidly varying fine structure has been “averaged out” to reveal the more slowly varying distortion component (blue line in Fig. 8). The reflection component can now be obtained by vector subtraction. This procedure for unmixing the distortion and reflection components of the total DPOAE, known as spectral smoothing [42–44], is the frequency-domain equivalent of separating the two components based on their respective latencies. Although the optimal unmixing procedure has yet to be definitively established, methods based on spectral smoothing, on time-frequency filtering using continuous wavelet transforms, or on the use of additional stimulus tones (or sweeps) strategically placed to reduce the reflection component via nonlinear suppression all generally yield similar results, at least when applied in normal-hearing and/or simulated ears [42, 45–47]. With the exception of the suppression method, all require data with high frequency resolution.

## Up or Down?

When employing linear, exponential, or other monotonic sweeps one must decide how to set the switch that controls sweep direction, up or down. As illustrated in Fig. 4, bats have generally chosen to sweep down. Perhaps this is because down-sweeps help to compensate for traveling-wave dispersion, thereby reducing intracochlear suppression and masking [48–51].

**Fig. 9:**
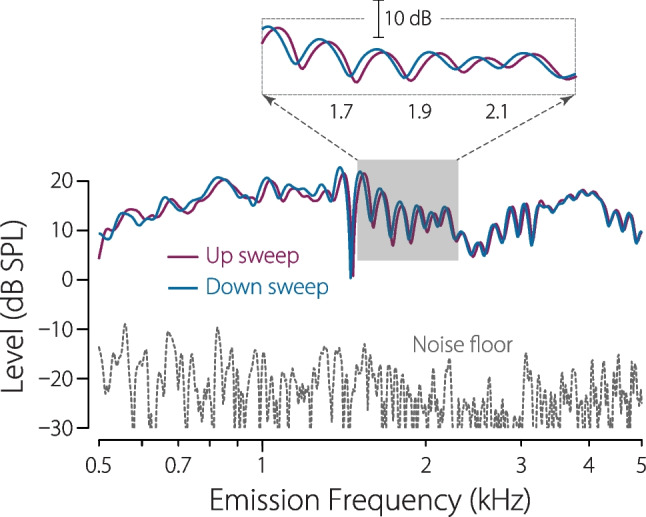
Frequency shifts in DPOAE fine structure. The figure shows the DPOAE levels at $2f_1-f_2$ evoked from another human ear using $f_1$ and $f_2$ primary tones swept together, first in one direction and then in the other (e.g., up then down). DPOAEs evoked by up-sweeps are shown in purple and down-sweeps in blue. The inset zooms on the shaded region (1.5–2.3 kHz) to show a band of pronounced fine structure in more detail. The measurement noise floor averages around $-20$ dB SPL. Sweep parameters: $f_2/f_1=1.22$; $\{L_1,L_2\}=\{65,65\}\,$ dB SPL; rates: $\pm 2\,$ oct/s. Adapted from [26]

But does sweep direction make any difference when measuring DPOAEs with swept tones? It turns out that when the sweep rates are sufficiently slow (that is, less than about 1 oct/s), sweep direction has little effect. Up- and down-sweeps give almost identical results, and both are nearly indistinguishable from the DPOAEs obtained using discrete tones. At higher sweep rates, however, subtle differences between up and down become apparent [26, 52, 53]. Figure 9 compares the DPOAEs measured using up- and down-sweeps presented at $\pm 2$ oct/s, respectively. The only obvious difference—a difference significantly larger than the typical test-retest variability—is the systematic shift in the fine-structure pattern. As highlighted in the inset, the fine structure shifts upwards for the up-sweep and downwards for the down-sweep.

Shifts occur because the two components (reflection and distortion) that interfere to produce the fine-structure pattern have different latencies (phase-gradient delays) [26]. As a result, when evoked by swept tones, the component pressures that mix at the microphone at any instant of time have slightly different frequencies. When the measured waveform is subsequently analyzed using the least-squares procedure (which typically takes no account of the two different frequencies involved), this difference causes an apparent shift in fine structure. The magnitude of the shift is proportional to the sweep rate times the difference in component latency [26].

## Advantages and Applications

Swept-tone measurements offer a number of advantages over traditional discrete-tone measurements. They provide high frequency resolution, resolution that can even be changed after the fact by reanalyzing the measured waveforms. Among other benefits, the high frequency resolution facilitates the computation of phase-gradient delays. Accurate characterization of these delays facilitates the post hoc separation of DPOAEs into distortion and reflection components using offline signal-processing methods.

**Fig. 10:**
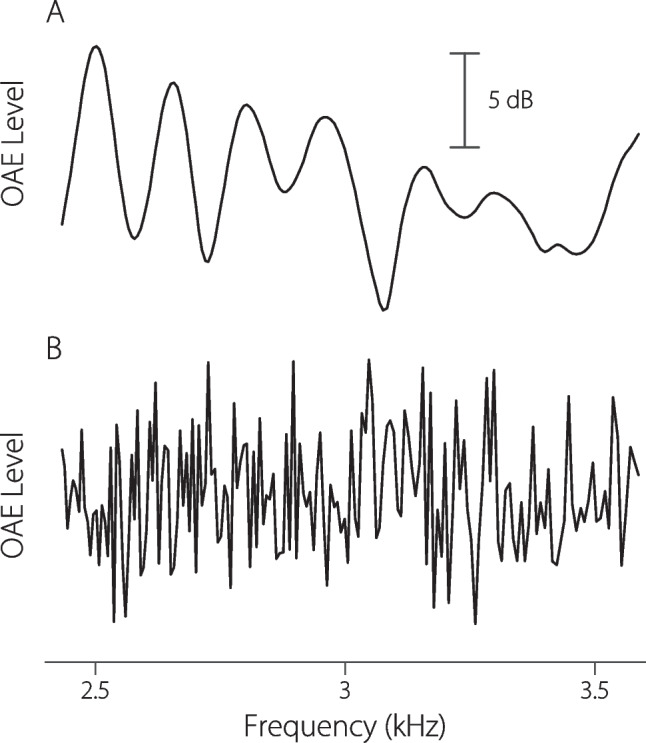
Real and hypothetical OAE spectra. Panel **A** shows a typical human DPOAE amplitude spectrum in which OAE levels at nearby frequencies are strongly correlated with one another. The amplitude variation seen here arises both from quasi-periodic interference between the distortion and reflection components (fine-structure) as well as possible intrinsic spatial variations in OAE source strength. Panel **B** shows a hypothetical spectrum in which OAE amplitudes at different frequencies are essentially independent of one another. Although the spectrum looks irregular and “noisy,” the OAE levels are imagined to be as reproducible as those in panel A

When high-resolution data are desired, using sweeps rather than discrete tones also improves the efficiency of data collection. To see this, note that sweeps analyzed with the least-squares method allow one to exploit the fact that OAE levels at nearby frequencies are correlated with one another but the noise is not. Consequently, swept-tone methods can expedite high-resolution measurements without prolonging the total measurement time or sacrificing the SNR. If OAE levels varied with frequency as illustrated in Fig. 10B—that is, if they varied irregularly but reproducibly from one frequency to the next, like the frequency response of a highly reverberant room—then analysis bandwidths would need to be kept narrow (e.g., by using discrete tones) to capture the detailed shape of the spectrum. But OAE levels do not look anything like this; rather, they look like the smoother spectrum shown in Fig. 10A.^5^ OAE levels vary smoothly with frequency because they arise from a relatively broad region of the cochlea determined by the width of the traveling-wave envelope, whose spatial averaging smooths out any irregularities the ear’s speech might have [55]. Consequently, by sweeping the stimulus frequency and widening the analysis bandwidth to be commensurate with the correlation bandwidth, one can cover a broader range of frequencies in the same measurement time. (At each frequency, the effective measurement “dwell time” [10, 56] is roughly $\textrm{BW}/|r|$, where $\textrm{BW}$ is the analysis bandwidth and *r* is the sweep rate.) In a nutshell, one can quickly obtain high-resolution measurements because, unlike when using discrete tones, time spent measuring at one frequency also helps lower the noise at others.^6^

**Fig. 11:**
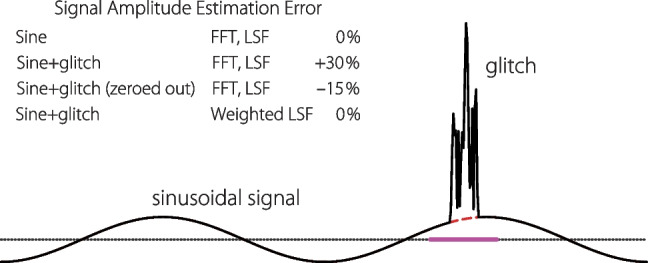
Benefits of weighted least squares illustrated using a sinusoidal signal with an optional glitch (artifact). When the signal is artifact-free, both the fast Fourier transform (FFT) and regular (unweighted) least-squares fitting (LSF) yield accurate estimates of the signal amplitude. However, when the glitch is present—or identified and simply zeroed out over the region identified by the purple bar along the dotted zero line—both methods yield significant estimation errors. By contrast, using weighted LSF (with weights in the glitch region set to 0) restores the accuracy obtained for the waveform without artifacts

Although other analysis methods with their own special utility are available (e.g., inverse filtering or time compression [27, 57]), one significant advantage of analyzing sweep waveforms using the least-squares method is the ability to control the relative weighting of different samples (or time segments) when analyzing the measured response waveforms. For example, by using weighted least squares one can remove the contaminating influence of intermittent artifactual glitches that occur during the recording (e.g., because of subject movements or environmental noise). (One could, of course, simply discard the entire response waveform and proceed to measure another, desperately hoping to avoid another glitch. But this takes time and inefficiently throws the otoacoustic baby out with the glitchy bathwater.) By identifying glitches in the waveform, and setting the corresponding weights to zero, one can effectively eliminate these samples from the analysis without compromising the accuracy of the results. Figure 11 provides a simple example, illustrating the benefits of weighted least squares for signal estimation by comparing its results to those of standard Fourier analysis, which offers no such flexibility.

The swept-tone method was first introduced as a convenient way of exploring DPOAE fine structure and its modulation by efferent feedback by unmixing the total OAE into “generator” and “reflection” components [23, 58, 59]. Since then, swept-tone methodology has been advanced [10, 14, 53] and extended to stimulus-frequency OAEs [25, 60–64]. The method enables the efficient measurement of both high-resolution distortion and reflection emissions in the same subjects [65] and of OAE frequency spectra and phase-gradient delays at multiple stimulus levels, from which input/output functions at specific frequencies can later be constructed offline [66].

Swept-tone methods have been applied in a diverse array of ears, including both the normal and the hearing impaired [67], the latter from a variety of etiologies [68]; in humans to study the maturation and aging of the peripheral auditory system in subjects ranging from newborns to the elderly [69–73]; in young adults to study the breaking of scaling symmetry [32, 33, 35]; and in comparative studies involving other animals, including mice, gerbils, anole lizards, barn owls, and clouded leopards [74, 75]. The swept-tone method also provides a valuable tool for probing cochlear mechanics, especially the complex temporal interactions between nonlinearity and dispersion [37, 76]. Indeed, by helping to interpolate between clicks and tones, swept-tone stimuli facilitate the study of both physiological and behavioral responses to dynamic sounds. As an example of the unexpected insights obtained, swept-tone measurements of reflection- and distortion-OAE phase spanning 5 octaves in frequency and a wide range of primary frequency ratios ($f_2/f_1$) suggest that the traditional division of the human cochlea into two broad regions—apical and basal—may be incomplete [34].

Although swept-tone methodology has clearly been a boon to research, realizing its potential contribution to the clinical assessment of hearing awaits the completion of ongoing studies [77]. Perhaps the goal of exploiting the power and flexibility of swept tones to enhance the clinical utility of otoacoustic emissions for the detection and/or differential diagnosis of hearing loss may finally be coming within reach.

### Supplementary Information

Below is the link to the electronic supplementary material.

Supplementary file 1 (pdf 219 KB)

Supplementary file 2 (mp4 1092 KB)

Supplementary file 3 (mp4 325 KB)

## Footnotes

1 At the same 2004 Midwinter Meeting of the ARO during which swept-tone DPOAEs made their debut [5], and presented almost side-by-side, just two posters down the aisle, Stephen Neely and his colleagues described a method for continuously varying the primary levels [6–8].

2 To appreciate the depth of the times involved, note that the scale along the abscissa in Fig. 4 indicates how twice the expected SNR for a synchronous averaging task varies with the measurement time. Thus, relative to the SNR achieved during the first year, measuring a sinusoid embedded in Gaussian noise for the full 65 million years since the K-T extinction lowers the noise floor by some $\frac{1}{2}20\log _{10}(65) = 78\,$ dB.

3 Although echolocation evolved multiple times on Earth—not only in bats but also in toothed whales (e.g., dolphins), as well as in shrews and tenrecs [18], oilbirds and swiftlets [19], and some blind humans [20]—only laryngeal bats employ frequency-modulated, chirp-like signals rather than acoustic clicks.

4 To estimate the group velocity of the traveling wave, we approximate both the BM frequency response ($\textrm{TF}$) and the traveling-wave envelope ($\textrm{TW}$) as Gaussians of widths $\sigma _f$ and $\sigma _x$, respectively. Thus, $\textrm{TF}= \exp [-\frac{1}{2}((f-\textrm{CF})/\sigma _f)^2]$ and $\textrm{TW}= \exp [-\frac{1}{2}((x-\hat{x})/\sigma _x)^2]$, where $\textrm{CF}$ is the characteristic frequency and $\hat{x}$ is the best place (peak of the wave). When the cochlear tonotopic map is exponential, the widths $\sigma _f$ and $\sigma _x$ are related by $\sigma _x=(\ell /\textrm{CF})\sigma _f$, where $\ell$ is the exponential space constant of the map. The equivalent rectangular bandwidth ($\textrm{ERB}$) of the Gaussian filter is given by $\textrm{ERB}=\sqrt{\pi }\sigma _f$. We approximate the group velocity at the peak of the traveling wave by $v_\textrm{g}\approx \Delta x/\tau$, where $\Delta x$ is the effective width of the peak region and $\tau$ is the wave travel time across this region. Since OAE latency is dominated by round-trip delays within the peak region, we approximate $\tau$ as $\tau \approx \frac{1}{2}\tau _\textrm{SFE}$, where $\tau _\textrm{SFE}$ is SFOAE group delay. Approximating the width of the scattering region as $\Delta x\approx 4\sigma _x$, we have $\Delta x\approx 4\ell \sigma _f/\textrm{CF}= 4\ell /(\sqrt{\pi }Q_{\textrm{ERB}})$, where $Q_{\textrm{ERB}}=\textrm{CF}/\textrm{ERB}$. Putting it all together yields $v_\textrm{g}=8\ell \textrm{CF}/(\sqrt{\pi }N_\textrm{SFE}Q_{\textrm{ERB}})$, where $N_\textrm{SFE}=\tau _\textrm{SFE}\textrm{CF}$ is SFOAE delay in stimulus periods. Relating $N_\textrm{SFE}$ and $Q_{\textrm{ERB}}$ using the tuning ratio ($Q_{\textrm{ERB}}=rN_\textrm{SFE}$) yields $v_\textrm{g}=8\ell \textrm{CF}/(\sqrt{\pi }rN_\textrm{SFE}^2)$. To relate this velocity to sweep rates it is convenient to express $v_\textrm{g}$ in octaves/s (rather than, say mm/s). For this, we use the relation $\Delta x_\textrm{oct}=\Delta x/(\ell \ln 2)$. Hence, $v_\textrm{g}=8\textrm{CF}/(\sqrt{\pi }\ln 2\,rN_\textrm{SFE}^2)$ (oct/s). Figure 5 is computed using published human SFOAE delays [29] and averaged tuning ratios for cat, guinea pig, and chinchilla [30, 31].

5 The figure shows human DPOAE data. In common laboratory animals, correlation bandwidths are typically a factor of 2 or 3 larger. This is true not only because animal DPOAE fine-structure patterns vary more slowly with frequency (since their OAE delays are shorter than those of humans [54]), but also because the peak-to-peak amplitude of the fine-structure oscillations is generally also reduced.

6 For a fixed SNR, the potential reduction in measurement time depends on the desired frequency resolution of the measurement. If this resolution is $\Delta f$ and the chosen analysis bandwidth is $\textrm{BW}$ (with $\textrm{BW}>\Delta f$), then the use of swept tones can speed up the total measurement by roughly a factor of $\frac{1}{2}(\textrm{BW}/\Delta f)$. If $\Delta f>\frac{1}{2}\textrm{BW}$ then one may be better off using discrete tones.
