# Supplementary material for: Swept Along: Measuring Otoacoustic Emissions Using Continuously Varying Stimuli
Source: J Assoc Res Otolaryngol. 2024 Feb 26;25(2):91–102. doi: 10.1007/s10162-024-00934-5 (PMC11018600; doi:10.1007/s10162-024-00934-5)
Supplement: Supplementary file 1 — (pdf 219 KB) [file 10162_2024_934_MOESM1_ESM.pdf]

SUPPLEMENTARY INFORMATION

Journal of the Association for Research in Otolaryngology

Swept Along: Measuring Otoacoustic Emissions Using Continuously Varying Stimuli

Christopher A. Shera

Auditory Research Center
Caruso Department of Otolaryngology and Department of Physics & Astronomy
University of Southern California, Los Angeles, California, USA
apg.mechanicsofhearing.org; christopher.shera@usc.edu

Caption for the Lorenz Attractor Video (Online Resource 2)

The supplemental video and its soundtrack[1] were created by solving the coupled system of Lorenz equations [1],

$$\frac{d}{dt}\begin{bmatrix}x\\y\\z\end{bmatrix} = \begin{bmatrix}\sigma(y-x)\\x(\rho-z)-y\\xy-\beta z\end{bmatrix} , \tag{1}$$

using standard parameters ($\{\sigma,\rho,\beta\} = \{10, 28, 8/3\}$) and initial conditions ($\{x,y,z\}_0 = \{20, 5, -5\}$). The equations were solved numerically using the Runge-Kutta method [2] (implemented as `ode23` in MATLAB) on the time interval $[0, 36]$ s using a sampling rate of 16 kHz. The instantaneous frequency trajectories, $f_1(t)$ and $f_2(t)$, were obtained from the solutions $x(t)$ and $z(t)$ using the equations

$$\begin{bmatrix}f_1(t)\\f_2(t)\end{bmatrix} = f_0\begin{bmatrix}2^{x(t)/30}\\\frac{5}{2}\,2^{[z(t)-25]/30}\end{bmatrix} , \tag{2}$$

with $f_0 = 600\,\text{Hz}$. Finally, the stimulus waveforms were computed as

$$\begin{bmatrix}s_1(t)\\s_2(t)\end{bmatrix} = \sin\left(2\pi\int_0^t\begin{bmatrix}f_1(t')\\f_2(t')\end{bmatrix}dt'\right) . \tag{3}$$

The two waveforms were subsequently summed, tapered at onset and offset, and sped up by a factor of 3 for monaural playback. The video is therefore $36/3 = 12\,\text{s}$ in duration when played at the sampling rate of $3\cdot 16 = 48\,\text{kHz}$.

Caption for the Lissajous Audio File (Online Resource 3)

The supplemental audio file was created by first defining the following functions

$$f_{\text{DP}}(t) = f_{\text{DP}_1}\left(\frac{f_{\text{DP}_2}}{f_{\text{DP}_1}}\right)^{\Lambda(2\pi N_{\text{DP}}t/T)} , \tag{4}$$

and

$$r(t) = r_1\left(\frac{r_2}{r_1}\right)^{\Lambda(2\pi N_r t/T)} . \tag{5}$$

In these equations, the parameters $f_{\text{DP}_{\{1,2\}}}$ represent the desired minimum and maximum values, respectively, of the DP frequency, $f_{\text{DP}} = 2f_1 - f_2$. Similarly, the parameters $r_{\{1,2\}}$ represent the desired range of primary frequency ratios, $r = f_2/f_1$. The parameters N_{DP} and N_r specify the desired number of complete Lissajous "orbits" traversed during the time T, where T is the total stimulus duration. The function $\Lambda(t)$ represents a sawtooth waveform in sine phase with range $[0, 1]$ and period 2π. For example,

$$\Lambda(t) = \frac{1}{2}\left\{1 + \frac{2}{\pi}\sin^{-1}[\sin(t-\pi/2)]\right\} . \tag{6}$$

[1] The supplemental video and audio files can also be found on the Auditory Physics Group website:

- `apg.mechanicsofhearing.org/downloads/sounds/Swept-Along-Lorenz.mp4`
- `apg.mechanicsofhearing.org/downloads/sounds/Swept-Along-Lissajous.mp4`

With these definitions, the instantaneous frequencies $f_1(t)$ and $f_2(t)$ were obtained using the equations

$$f_2(t) = \frac{f_{\mathrm{DP}}(t)}{2/r(t) - 1} \ , \tag{7}$$

and

$$f_1(t) = f_2(t)/r(t) \ . \tag{8}$$

The stimulus signals were then computed as

$$\begin{bmatrix} s_1(t) \\ s_2(t) \end{bmatrix} = \sin \left(2\pi \int_0^t \begin{bmatrix} f_1(t') \\ f_2(t') \end{bmatrix} dt' \right) \ . \tag{9}$$

The two stimulus signals were subsequently sampled at 48 kHz using the parameters $f_{\mathrm{DP}_{\{1,2\}}} = \{0.5, 2\}\,\mathrm{kHz}$, $r_{\{1,2\}} = \{1, 4\}$, $N_{\mathrm{DP}} = 5$, $N_r = 9.5$, and $T = 20\,\mathrm{s}$. With these parameters, the equivalent instantaneous sweep rate is $\pm 1\,\mathrm{oct/s}$ at the DP frequency. Finally, the resulting waveforms were summed for monoaural playback and tapered at onset and offset.

References

[1] Lorenz EM (1963) Deterministic nonperiodic flow. J Atmos Sci 20:130–141

[2] Forsythe GE, Malcolm MA, Moler CB (1977) *Computer Methods for Mathematical Computations.* Prentice Hall: Englewood Cliffs, New Jersey
